# Prevalence of insomnia and anxiety among healthcare workers during the COVID-19 pandemic in Jilin Province

**DOI:** 10.1590/1414-431X2020e10602

**Published:** 2021-06-14

**Authors:** Han-shuo Dong, Jing-jing Gao, Yu-Xiang Dong, Chun-xia Han, Li Sun

**Affiliations:** 1 Department of Geriatrics, Jilin Academy of Chinese Medicine Sciences, Changchun, Jilin, China; 2 Center of Preventive Treatment of Diseases, the Affiliated Hospital to Changchun University of Chinese Medicine, Changchun, Jilin, China; 3 Department of Traditional Chinese Medicine, The First Hospital of Jilin University, Changchun, Jilin, China; 4 Department of Acupuncture and Massage, Jilin Academy of Chinese Medicine Sciences, Changchun, Jilin, China

**Keywords:** Anxiety, Insomnia, COVID-19, Healthcare workers, HCWs

## Abstract

The outbreak of the 2019 novel coronavirus disease (COVID-19) has impacted the mental health of healthcare providers at the frontline. Therefore, we conducted this study to estimate the prevalence rate of anxiety and insomnia and identify associated risk factors among healthcare workers in Jilin, China, during the period from January 25 to February 25, 2020. Zung's Self-Reported Anxiety Scale (SAS) and the Insomnia Severity Index (ISI) scale were used to diagnose anxiety and insomnia, respectively. Associated risk factors were identified through a multivariate logistic regression model. A total of 300 healthcare workers were invited and 236 completed the study. Of them, 234 (99.15%) were medical workers, 197 (83.47%) were working at frontline departments, and 159 (67.37%) were fighting against COVID-19. Fifty-seven respondents (24.15%) had anxiety (SAS index score ≥45) and 94 (39.83%) had insomnia (ISI score ≥8). Based on the multivariate analysis, contact with people from Hubei province during work (no *vs* not clear) [OR=0.25, 95%CI: 0.10-0.61] and personal protective equipment (PPE) (not in place *vs* in place) [OR=6.22, 95%CI: 2.23-17.40] were significantly correlated with anxiety. PPE (not in place *vs* in place) was the only significant risk factor of insomnia [OR=10.56, 95%CI: 4.00-27.87]. The prevalence of anxiety and insomnia was high in our study, reflecting the psychological impact of COVID-19 on healthcare workers. The unavailability of PPE in place was a significant risk factor of both anxiety and insomnia.

## Introduction

The 2019 novel coronavirus disease (COVID-19) was first reported in Wuhan, Hubei Province, China, and within a short period of time, the disease was recognized as a major public health concern all over the globe (1). In response to this devastating disease, the Chinese health department deployed over 30,000 medical personnel from other provinces to assist in fighting against the outbreak of COVID-19 in Hubei and to prevent its dissemination outside Hubei province (1). As of May 3, 2020, confirmed cases of COVID-19 were no longer limited to China, and the disease was reported in 212 other countries and territories (2). Furthermore, the overall number of confirmed cases has now reached over 3.5 million worldwide (2).

The outbreak of COVID-19 has not only affected the preparedness of many healthcare systems (3), but it has also substantially impacted the psychological stability of many healthcare and non-medical workers who are at the frontline fighting against COVID-19 (4). Such frontline workers are at significant risk of developing serious psychological distress as well as many other mental health problems. This is secondary to the increase in workload and media coverage, reduction and depletion of personal protective equipment (PPE), and the feelings of being improperly supported.

Therefore, many local and national Chinese mental health institutions have deployed various psychological assistance services, which were either telephone-, internet-, or application-based. Moreover, the State Council of China announced on February 2, 2020 that it was setting up hotlines aimed at providing psychological support during the current pandemic (5). That being said, the available body of evidence on the magnitude of mental health problems and correlated factors among healthcare workers (HCWs) who are taking care of patients exposed to COVID-19 remains very limited. A recent cross-sectional study of 1257 healthcare workers fighting against COVID-19 in 34 hospitals in China revealed that a high proportion of healthcare workers suffer from anxiety (44.6%) and insomnia (34.0%) (4). The authors also noted that frontline workers who are in direct contact with COVID-19 patients are at increased risk of anxiety and insomnia. This study highlights the substantial impact of COVID-19 on the mental health of frontline healthcare workers.

Nevertheless, the number of reports investigating the psychological impact of COVID-19 among such workers remains scarce. Thus, we conducted the current study to determine the prevalence of insomnia and anxiety among frontline HCWs during the outbreak of COVID-19 at Jilin Province. We also aimed at estimating the various degrees of anxiety and insomnia among HCWs, as well as determining the significant determinants of anxiety and insomnia among them.

## Material and Methods

### Study design and sample size calculation

This cross-sectional survey-based study was conducted among healthcare personnel working at Jilin Province during the COVID-19 pandemic. All hospitals at Jilin Province were invited to participate in this study. During the period from January 25 to February 25, 2020, an online self-administered questionnaire was distributed among eligible participants, and filled questionnaires were retrieved and reviewed. The study protocol was approved by the Ethics Committee of the First Clinical Hospital of Jilin Academy of Chinese Medical Sciences Sleep Center at Jilin Province, China. Meanwhile, informed consent was collected from each eligible individual prior to participation in this study. All participants had the right to withdraw from the study at any time, and the confidentiality of all participants was assured. On the other hand, individuals who were unwilling to participate, those who were unable to understand the questionnaire, and had incomplete data were excluded.

The sample size of our study was estimated using the Raosoft software (6,7). With a margin of error of 5%, confidence level of 90%, and response rate of 68.7%, the proposed sample size was 231 participants, based on the study of Lai et al. (4). The target sample size of our study was estimated as 164 participants using the following formula: N = Z_α_^2^ P(1−P) / d^2^, in which α=0.005 and Z_α_=2.576, and the estimated acceptable margin of error for proportion d was 0.1. The proportion (P) of HCWs with anxiety was estimated at 44.6%, based on the previous report of Lai et al. (4). A total of 300 individuals were invited to participate in our study and 236 individuals were recruited.

### Measurement tools

Our primary outcome was to determine the prevalence rates of anxiety and insomnia among respondents, while our secondary outcome was to identify the significant risk factors of the primary outcomes. In order to do that, we used three questionnaires designed through a professional platform (http://wjx.cn) and distributed among participants through the WeChat/Weixin mobile application. The first questionnaire included 24 items related to baseline demographic characteristics and changes in lifestyles and working habits. These items included age, gender, educational level, marital status, job nature, working experience and workload, job title, whether the main task was to treat patients with COVID-19, history of contact with people from Hubei Province, history of exposure to COVID-19, worry about the risk of infection, living status, average time of discussing COVID-19-related information, and changes in sleep duration and quality, exercise duration and intensity, diet, and physical condition.

The second questionnaire was the Zung's Self-reported Anxiety Scale (SAS). This scale included 20 items, covering a wide range of anxiety symptoms, both psychological (i.e., “I feel afraid for no reason at all” and “I feel like I'm falling apart and going to pieces”) and somatic (i.e. “My arms and legs shake and tremble” and “I feel my heart beating fast”) in nature. Responses were recorded on a 4-point Likert-type scale, which ranges from 1 (none, or a little of the time) to 4 (most, or all of the time). Surveyed participants were instructed to base their responses on their experiences during the previous week (Supplementary Figure S1). SAS items included both negative (i.e., “I feel more nervous and anxious than usual”) and positive (i.e., “I fall asleep easily and get a good night's sleep”) experiences, while the latter was reverse scored. Each participant had a raw score ranging from 20 to 80. These scores were later multiplied by 1.25 to determine the index score of (25-100). Overall, anxiety was defined with an index score of 50 (cut-off value) (8,9). Mild, moderate, and severe anxiety were defined with index scores of 45-59, 60-74, and 75-100, respectively.

The third questionnaire was the Insomnia Severity Inventory (ISI) scale, which was used to measure the prevalence and severity of insomnia among surveyed workers within the past two weeks (10). The ISI scale is composed of 7 items, and each item is given a score of 0-4 on a Likert scale, with a total score ranging from 0 to 28. A cut-off value of 8 was used to define insomnia (10,11). Mild, moderate, and severe insomnia were classified based on scores of 8-14, 15-21, and 22-28, respectively (Supplementary Figure S2).

All participants were asked to complete the study questionnaires only once, with no follow-up questionnaires being distributed after that point.

### Statistical analysis

All questionnaires with complete data were entered into a standardized Excel sheet and then analyzed using SAS (Version 9.2, USA) software. Using the chi-squared test or Fisher test, a simple descriptive statistic was used to define the characteristics of the study variables in the form of number and percentage for categorical variables and mean and standard deviation (SD) for continuous variables. The non-parametric Mann-Whitney U test and Kruskal-Wallis test were used to compare the severity of the outcomes (anxiety or insomnia) between two or more groups. In order to identify the potential risk factors of anxiety and insomnia, we designed a univariate regression model. Significant independent variables, based on the univariate model, were then incorporated into a multivariate logistic regression model to control for potential confounders. The correlation between the independent variables and the outcomes (dependent variables) are reported as odds ratios (ORs) and the corresponding 95% confidence interval (CI). A P-value of <0.05 was used as the cut-off point of statistical significance.

## Results

### Baseline characteristics of respondents

A total of 300 HCWs were invited to participate in our study and 236 participants (78.67%) completed the study and were included in the analysis. Our population had a mean age of 35.37±10.1 years. The majority of respondents were females (97.88%), married (66.1%), medical workers (99.15%), working in the frontline department (83.47%), with 0-5 years of experience (38.98%). The main task of the majority of recruited HCWs was to fight against COVID-19 (67.37%); however, a minimal number of participants were exposed to suspected/confirmed cases or specimens of COVID-19 during work (22.03%). In addition, 91.1% of respondents reported receiving enough training for protection against COVID-19, while 47.46% reported that PPE was ‘basically in place’ in the workplace. Less than half of respondents (31.78%) reported being worried about the increased risk of becoming infected with COVID-19 during work. Sleep duration and quality were worse in 38.13% and 45.34% of respondents, respectively. Baseline demographic and occupational characteristics of recruited participants are reported in detail in Supplementary Table S1.

### Prevalence of anxiety and associated factors

Overall, a total of 57 (24.15%) respondents suffered from anxiety; all of them were female HCWs. Forty respondents had mild anxiety, 14 had moderate anxiety, and 3 had severe anxiety. The differences between the various degrees of severity of anxiety are reported in Supplementary Table S2. Based on the minimal number of respondents having moderate and severe anxiety, we combined all degrees of anxiety into a single variable (anxiety, no anxiety) for the purposes of identifying the correlating risk factors.

Those who were in contact with people from Hubei during the pandemic (25 *vs* 18.75%; P<0.001) and those who were exposed to suspected or confirmed cases of COVID-19 (26.92 *vs* 19.50%; P=0.007) were significantly more likely to have anxiety compared to those with no prior contact or exposure, respectively. PPE ‘not in place' resulted in a significantly higher rate of anxiety compared to PPE ‘in place’ (60.71 *vs* 13.54%; P<0.001). HCWs who were worried about the increased risk of infection with COVID-19 were significantly more likely to have anxiety compared to unworried personnel (37.33 *vs* 10.17%; P=0.001). Worsened sleep duration (42.22 *vs* 28.57%; P<0.001) and sleep quality (40.19 *vs* 0%; P<0.001) were significantly associated with higher anxiety rates compared to better sleep outcomes, respectively. Similarly, worsened appetite (48.39 *vs* 9.09%; P<0.001) and diet regularity (44.59 *vs* 11.11%; P=0.002) were significantly associated with higher anxiety rates, respectively. In the same context, shortened exercise duration (33.58 *vs* 30%; P<0.001) and weakened exercise intensity (32.33 *vs* 28.57%; P=0.002) were also associated with significantly higher anxiety rates, respectively. Finally, those with poor ‘sub-health' physical conditions were significantly more likely to suffer from anxiety compared to those with a good physical condition (43.37 *vs* 9.42%; P<0.001) (Supplementary Table S3).

Female gender (P=0.017), married status (P=0.021), contact with people from Hubei during the pandemic (P<0.001), exposure to suspected and/or confirmed COVID-19 cases (P<0.001), worry about increased risk of being infected with COVID-19 (P<0.001), PPE ‘not in place' at work (P<0.001), worse sleep duration and quality (P<0.001), worse appetite (P<0.001) and diet regularity (P<0.001), shortened exercise duration (P<0.001) and weakened exercise intensity (P=0.001), and poor physical condition (P<0.001) were significantly correlated with higher mean anxiety scores (Supplementary Table S4).

Based on the single-factor logistic regression analysis, we found that contact with people from Hubei province during the pandemic (OR=4.64; 95%CI: 1.19-18.10], PPE ‘not in place' at work [OR=9.87; 95%CI: 3.79-25.70], worry about increased risk of COVID-19 infection [OR=0.19; 95%CI: 0.07-0.50], and increase in the time discussing COVID-19-related information [OR=2.32; 95%CI: 1.06-5.06] were significantly correlated with anxiety (Table 1). In the multivariate regression model, previous contact with people from Hubei during the pandemic [OR=0.25; 95%CI: 0.10-0.61] and the availability of PPE [OR=6.22; 95%CI: 2.23-17.40] were the only significant independent risk factors for anxiety (Table 2).

**Table 1:** Univariate logistic regression analysis of factors associated with anxiety in healthcare workers during the COVID-19 pandemic.

Variable	b	Standardized b	Wald χ^2^	P	OR	95%CI for OR	
						lower	upper
Gender	13.48	659.68	0.00	0.984	-	0.00	-
Age	0.00	0.02	0.01	0.914	1.00	0.97	1.03
Education level							
Junior college *vs* high school/secondary school	1.17	1.09	1.15	0.284	3.23	0.38	27.65
Undergraduate *vs* high school/secondary school	0.83	1.08	0.60	0.439	2.30	0.28	19.08
Master's degree *vs* high school	1.39	1.27	1.18	0.277	4.00	0.33	48.65
Marital Status							
Married *vs* unmarried	0.64	0.36	3.14	0.076	1.90	0.93	3.88
Divorced *vs* unmarried	1.64	1.05	2.45	0.117	5.17	0.66	40.34
Widowed *vs* unmarried	1.64	1.45	1.28	0.257	5.17	0.30	88.42
Occupation							
Other *vs* medical work	1.16	1.42	0.66	0.416	3.18	0.20	51.65
Department							
Executive *vs* frontline	-13.57	1043.05	0.00	0.990	0.00	0.00	-
Logistics department *vs* frontline	-13.57	1043.05	0.00	0.990	0.00	0.00	-
Other *vs* frontline departments	-0.77	0.51	2.26	0.132	0.46	0.17	1.26
Working years							
6-10 *vs* 0-5	0.48	0.47	1.01	0.315	1.61	0.64	4.06
11-15 *vs* 0-5	0.68	0.44	2.40	0.121	1.97	0.84	4.66
16-20 *vs* 0-5	0.72	0.67	1.17	0.279	2.06	0.56	7.59
21 and above *vs* 0-5	0.16	0.40	0.16	0.688	1.17	0.54	2.58
Job title							
Primary *vs* none	-0.17	0.42	0.17	0.681	0.84	0.37	1.92
Intermediate *vs* None	0.59	0.48	1.52	0.218	1.80	0.71	4.59
Mid-high or high *vs* none	-0.49	0.52	0.87	0.352	0.62	0.22	1.71
Have you come into contact with people from Hubei epidemic area in your work?							
No *vs* yes	-0.37	0.61	0.37	0.544	0.69	0.21	2.27
Not sure *vs* yes	1.53	0.70	4.87	0.027	4.64	1.19	18.10
Have you been exposed to suspected or confirmed cases or suspected/confirmed case specimens at work?							
No *vs* yes	-0.42	0.37	1.28	0.258	0.66	0.32	1.36
Not sure *vs* yes	0.92	0.51	3.27	0.071	2.51	0.93	6.78
Have you received enough training for the new coronavirus protection?							
No *vs* yes	-0.34	0.80	0.18	0.674	0.71	0.15	3.41
Not sure *vs* yes	0.76	0.67	1.31	0.252	2.14	0.58	7.89
Are protective measures in place at work?							
Basically in place *vs* in place	0.71	0.37	3.63	0.057	2.03	0.98	4.20
Not in place *vs* in place	2.29	0.49	21.95	0.000	9.87	3.79	25.70
Are you worried about increased risk of infection because of work?							
No *vs* yes	-1.66	0.49	11.37	0.001	0.19	0.07	0.50
A little bit *vs* no	-0.72	0.34	4.53	0.033	0.49	0.25	0.94
Day shift							
Yes *vs* No	0.42	0.31	1.86	0.173	1.52	0.83	2.78
Your working hours in the last two weeks, compared to usual							
Increased *vs* no change	0.60	0.44	1.84	0.175	1.83	0.76	4.37
Decreased *vs* no change	-0.11	0.35	0.11	0.744	0.89	0.45	1.77
Living status in the last two weeks							
Living with family *vs* living alone	0.26	0.37	0.48	0.488	1.29	0.62	2.68
Living with friends and colleagues *vs* living alone	-0.17	0.72	0.05	0.816	0.85	0.21	3.46
Living with others *vs* living alone	-13.29	851.65	0.00	0.988	0.00	0.00	
Did you take the initiative to isolate your family because of your work?							
Yes vs no	-0.17	0.31	0.28	0.597	0.85	0.46	1.57
In the past two weeks, the average time you discussed the epidemic information every day (hours)							
1-2 *vs* less than 1	0.84	0.40	4.46	0.035	2.32	1.06	5.06
3-4 *vs* less than 1	0.51	0.53	0.91	0.340	1.66	0.59	4.73
5 and above *vs* less than 1	-0.60	0.82	0.54	0.462	0.55	0.11	2.73

OR: odds ratio; CI: confidence interval; P: P-value.

**Table 2:** Multivariate logistic regression analysis of factors associated with anxiety.

	b	Standardized b	Wald χ^2^	P	OR	95%CI for OR	
						lower	upper
Constant	-1.56	0.63	6.08	0.014			
Have you ever come into contact with people from Hubei Epidemic area?							
No *vs* yes	-0.45	0.62	0.52	0.473	0.64	0.19	2.17
Not sure *vs* yes	0.96	0.74	1.69	0.193	2.61	0.62	11.02
No *vs* not clear	-1.41	0.47	9.00	0.003	0.25	0.10	0.61
Protective measures in place at work							
Basically in place *vs* in place	0.67	0.38	3.15	0.076	1.96	0.93	4.10
Not in place *vs* in place	1.83	0.52	12.15	0.001	6.22	2.23	17.40

OR: odds ratio; CI: confidence interval; P: P-value.

### Prevalence of insomnia and associated factors

Based on the Insomnia Severity Index scale, the prevalence of insomnia in our population was 39.83% (94 cases). Seventy-four HCWs had mild insomnia, 15 had moderate insomnia, and 5 had severe insomnia. The differences between the various degrees of severity of insomnia are presented in Supplementary Table S5. Based on the minimal number of respondents having moderate and severe insomnia, we combined all degrees of insomnia into a single variable (insomnia, no insomnia) for the purpose of identifying the correlated risk factors.

Surprisingly, married people were more likely to suffer from insomnia compared to unmarried ones (42.95 *vs* 29.73%, P=0.007). Workers who came in contact with people from Hubei province during work were significantly more likely to have insomnia compared to others (43.75 *vs* 34.90%, P=0.001). Moreover, the presence of PPE ‘in place’ was associated with a reduced likelihood of insomnia compared to ‘not in place' (29.17 *vs* 64.29%, P=0.003). In the same context, people who were worried about the increased risk of COVID-19 infection were more likely to have insomnia compared to non-worried individuals (50.67 *vs* 22.03%, P=0.003). Other factors, such as poor/worse sleep duration and quality, appetite, diet regularity, exercise duration and intensity, and physical conditions significantly increased the likelihood of having insomnia (P<0.001) (Supplementary Table S6). All of the above factors resulted in statistically significant differences in mean insomnia severity scores (P<0.05) (Supplementary Table S7).

Based on the univariate regression model, we found that age [OR=1.03; 95%CI: 1.01-1.06], increased years of work experience (≥21 years) [OR=2; 95%CI: 1.04-3.87], job title [OR=2.95; 95%CI: 1.19-7.31], PEE ‘not in place' [OR=4.37; 95%CI: 1.80-10.64], no worry about the increased risk of COVID-19 infection [OR=0.28; 95%CI: 0.13-0.59], and increase in the time discussing COVID-19-related information (1-2 *vs* <1 h) [OR=2; 95%CI: 1.04-3.85] were significantly correlated with insomnia (Table 3). However, on further incorporation into a multivariate regression model, PPE was the only factor that remained significantly correlated with insomnia (P<0.001) (Table 4).

**Table 3:** Univariate regression analysis of factors associated with insomnia in healthcare workers during the COVID-19 pandemic.

	b	Standardized b	Wald χ^2^	P	OR	95%CI for OR	
						lower	upper
Gender	0.99	1.13	0.78	0.379	2.70	0.30	24.50
Age	0.03	0.01	6.00	0.014	1.03	1.01	1.06
Education level							
Junior college *vs* high school/secondary school	-0.72	0.72	1.00	0.317	0.49	0.12	1.99
Undergraduate *vs* high school/secondary school	-0.62	0.69	0.81	0.367	0.54	0.14	2.08
Master's degree *vs* high school	-0.92	0.97	0.88	0.347	0.40	0.06	2.70
Marital Status							
Married *vs* unmarried	0.58	0.30	3.66	0.056	1.78	0.99	3.21
Divorced *vs* unmarried	15.14	631.63	0.00	0.981	-	0.00	
Widowed *vs* unmarried	0.86	1.44	0.36	0.549	2.36	0.14	39.51
Occupation							
Other *vs* medical work	-13.87	884.64	0.00	0.987	0.00	0.00	-
Department							
Executive *vs* frontline	14.64	893.26	0.00	0.987	-	0.00	-
Logistics department *vs* frontline	-13.90	884.64	0.00	0.987	0.00	0.00	-
Other *vs* frontline departments	-0.42	0.39	1.15	0.283	0.66	0.30	1.41
Working years (years)							
6-10 *vs* 0-5	-0.06	0.44	0.02	0.887	0.94	0.40	2.23
11-15 *vs* 0-5	0.45	0.40	1.29	0.256	1.57	0.72	3.45
16-20 *vs* 0-5	1.06	0.63	2.88	0.090	2.89	0.85	9.87
21 and above *vs* 0-5	0.69	0.34	4.27	0.039	2.00	1.04	3.87
Job title							
Primary *vs* none	0.54	0.39	1.85	0.173	1.71	0.79	3.69
Intermediate *vs* none	1.08	0.46	5.44	0.020	2.95	1.19	7.31
Mid-high or high *vs* none	0.68	0.45	2.30	0.130	1.98	0.82	4.76
Have you ever touched people from Hubei epidemic area in your work?							
No *vs* yes	-0.37	0.53	0.50	0.479	0.69	0.25	1.93
Not sure *vs* yes	1.17	0.65	3.18	0.075	3.21	0.89	11.60
Were you exposed to suspected or confirmed cases or suspected/confirmed case specimens at work?							
No *vs* yes	0.16	0.33	0.23	0.634	1.17	0.61	2.24
Not sure *vs* yes	0.31	0.50	0.39	0.530	1.36	0.52	3.60
Have you received enough training for the new coronavirus protection?							
No *vs* yes	-0.56	0.69	0.65	0.422	0.57	0.15	2.22
Not sure *vs* yes	0.83	0.66	1.58	0.209	2.29	0.63	8.37
Are protective measures in place at work?							
Basically in place *vs* in place	0.60	0.29	4.14	0.042	1.82	1.02	3.25
Not in place *vs* in place	1.47	0.45	10.56	0.001	4.37	1.80	10.64
Are you worried about increased risk of infection because of work?							
No *vs* yes	-1.29	0.39	10.95	0.001	0.28	0.13	0.59
A little bit *vs* no	-0.34	0.31	1.26	0.262	0.71	0.39	1.29
Day shift							
Yes *vs* No	0.14	0.27	0.28	0.595	1.15	0.68	1.94
Working hours in the last two weeks, compared to usual							
Increased *vs* no change	0.33	0.42	0.63	0.428	1.39	0.61	3.17
Decreased *vs* no change	0.07	0.30	0.06	0.814	1.07	0.60	1.91
Living status in the last two weeks							
Living with family *vs* living alone	0.30	0.32	0.87	0.350	1.35	0.72	2.53
Living with friends and colleagues *vs* living alone	-0.51	0.64	0.63	0.426	0.60	0.17	2.11
Living with others *vs* living alone	-0.11	1.26	0.01	0.933	0.90	0.08	10.55
Did you take the initiative to isolate your family because of your work?							
Yes *vs* No	-0.03	0.27	0.01	0.905	0.97	0.57	1.65
In the past two weeks, the average time you discussed the epidemic information every day (hours)							
1-2 *vs* less than 1	0.69	0.33	4.29	0.038	2.00	1.04	3.85
3-4 *vs* less than 1	0.59	0.45	1.71	0.191	1.80	0.75	4.35
5 and above *vs* less than 1	0.41	0.53	0.59	0.441	1.50	0.53	4.24

OR: Odds Ratio; CI: Confidence Interval; P: P-value.

**Table 4:** Multivariate regression analysis of factors associated with insomnia in healthcare workers during the COVID-19 pandemic.

	b	Standardized b	Wald χ^2^	P	OR	95%CI for OR	
						lower	upper
Constant	-1.92	0.31	38.66	<.0001			
Protective measures in place at work							
Basically in place *vs* in place	0.79	0.38	4.29	0.038	2.20	1.04	4.63
Not in place *vs* in place	2.36	0.50	22.65	<.0001	10.56	4.00	27.87

CI: Confidence Interval; OR: Odds Ratio; P: P-value.

## Discussion

This cross-sectional, survey-based study initially investigated the prevalence of mental health symptoms (anxiety and insomnia) among 300 HCWs (236 respondents) who were involved in patient care during the outbreak of COVID-19 in Jilin Province, China. Due to the small number of respondents who reported moderate and severe symptoms, the analysis of associated factors based on the stratification of both outcomes (anxiety and insomnia) according to the severity of the symptoms was inapplicable.

The majority of our population consisted of female (97.88%) medical workers (99.15%), who were working in the frontline departments (83.47%) and were fighting against COVID-19 (67.37%). The prevalence of anxiety in our study was considerably high (24.15%), compared to the 5% weighted prevalence of anxiety disorders among the general population in China before the pandemic (12). We noted that HCWs, who are working in the frontline departments, who came in contact with people from Hubei Province, who were exposed to COVID-19-infected cases or specimens, who reported that PPE was ‘not in place', who were worried about the increased risk of COVID-19, and those who reported worse sleep duration/quality, worse diet appetite/regularity, poor exercise duration/intensity, and ‘sub-health' medical condition were significantly more likely to have anxiety compared to others. Since the outbreak of COVID-19 in China, many healthcare authorities have launched several web-based and telephone-based psychological assistance services to minimize the impact of such a pandemic on the mental health status of all individuals. However, it seems that such services were not able to significantly reduce the rates of mental health problems, particularly among frontline HCWs.

Several studies have studied the prevalence of mental health symptoms among medical workers during the COVID-19 pandemic. It was previously highlighted that females are significantly more likely to develop general anxiety disorder (4.1 *vs* 2.1%, OR=1.74; 95%CI: 1.37-2.22) (13). In our study, females were more likely to have a diagnosis of anxiety or insomnia; however, this difference was statistically non-significant. Also, gender was not a significant risk factor of either anxiety or insomnia in the regression models. This could be attributed to the fact that most of our study participants were females (97.88%). Therefore, more studies of nearly equal gender rates should further investigate this point.

Lai et al. (4) conducted a cross-sectional study among 1257 HCWs who were treating COVID-19 patients, at 34 hospitals in China (20 in Wuhan, 7 in other regions of Hubei province, and 7 hospitals from 7 other provinces), and they found that 44.6% of respondents had symptoms of anxiety based on the 7-item General Anxiety Disorder (GAD) scale. Similar to our study, the authors noted that frontline doctors were significantly more likely to suffer from anxiety. Furthermore, nurses, females, and medical personnel working at secondary hospitals and at Wuhan were also more likely to have anxiety. Moreover, Huang et al. (14) conducted a web-based questionnaire study among 7236 Chinese individuals during the pandemic of COVID-19. Of them, 2250 were HCWs, with a prevalence rate of anxiety of 37.37% (841 cases) based on the 7-item GAD scale. The authors reported that HCWs who spent more time (≥3h/day) thinking about COVID-19 were more likely to have anxiety compared to HCWs who spent less time. Similarly, Zhang et al. (15) reported a high prevalence rate of anxiety of 44.7% among 1563 HCWs, whose main task was to take care of patients during the COVID-19 outbreak. The authors also used the 7-item GAD scale to determine the rate of anxiety. On the other hand, other researchers reported much lower rates of anxiety among HCWs involved in patient care during the COVID-19 pandemic. In the study of Liu et al. (16), 512 medical workers from China completed an online questionnaire through WeChat, and 12.5% of them had symptoms of anxiety based on Zung's SAS questionnaire. Tan et al. (17) studied the prevalence of anxiety among a group of HCWs (470 respondents), who were working at 2 major tertiary hospitals that were involved mainly in taking care of patients with COVID-19. The authors reported a prevalence rate of anxiety of 14.5% based on the validated Depression, Anxiety, and Stress Scales (DASS-21). The prevalence rates of anxiety of the aforementioned observations and ours is inconsistent. This may be attributable to the use of different scoring scales. In the study that used the GAD scale, the prevalence rate of anxiety was higher (a cut-off value of 5 to define anxiety) (4,14,15), while the studies that used other scales (i.e., SAS) reported relatively lower anxiety rates (cut-off value of 50 to define anxiety) (16,17). Moreover, such differences can also be explained by the different settings at which each study was conducted; some hospitals may have been less affected by the COVID-19 pandemic, and thus, reflected fewer anxiety rates.

Based on the multivariate regression model, we found that only 2 variables were significant risk factors of anxiety in our study. A history of contact with people from Hubei during COVID-19 outbreak (no *vs* not clear) was associated with decreased risk of having anxiety (OR=0.25), while PPE (‘not in place’ *vs* ‘in place’) was associated with more than 6-times the risk of having anxiety (OR=6.22). Lai et al. (4) found that women (OR=1.69), secondary hospitals (OR=1.43), job title (intermediate) (OR=1.82), and frontline departments (OR=1.57) were significant risk factors associated with anxiety among HCWs treating COVID-19 patients. Meanwhile, Liu et al. (16) reported that medical personnel who had direct contact with infected individuals (OR=2.33) and those who were from Hubei province (OR=3.67) had a significantly higher risk of having anxiety, following the adjustment for sociodemographic characteristics (age, gender, educational level, and marital status).

The prevalence rate of insomnia was 39.83% among HCWs in our study. This is in agreement with the studies of Lai et al. (4) and Zhang et al. (15) who reported prevalence rates of insomnia of 34 and 36.1%, respectively. Both authors used the ISI questionnaire to define insomnia with a cut-off value of 8. This could have attributed to the similarity in our observations. Moreover, we found that increased age, marriage, frontline departments, contact with people from Hubei province, PPE (‘not in place’), worry about increased risk of infection, worse sleep duration/quality, worse diet appetite/regularity, poor exercise duration/intensity, and poor physical condition (sub-health) were associated with higher rates of anxiety compared to others. In the same context, Lai et al. (4) reported that nurses, women, frontline workers, tertiary hospitals, and doctors from Wuhan were more likely to have insomnia. The high prevalence of insomnia in the reported studies and in our study could be related to the increased stress that these HCWs face while treating patients with COVID-19. Such stress results in increased psychological and physical activation and the consistent activation of the hypothalamic-pituitary-adrenal (HPA) axis interferes with the normal sleep cycle (18). Subsequently, a sleeping disorder may occur, and it leads to further activation of the HPA axis, and thus, resulting in a vicious cycle of stress and insomnia (18).

In our study, we found that PPE was the only significant risk factor of insomnia based on the multivariate regression analysis. We noted the risk of insomnia was 10.56 times higher when PPE was ‘not in place' compared to when PPE was ‘in place'. Moreover, the frontline department was considered a significant risk factor of insomnia (OR=2.97) in another study that was conducted among medical workers fighting against COVID-19 (4). In another study, worry about being infected with COVID-19, perception of lack of proper psychological support, and extreme uncertainty of proper COVID-19 control measures were significant determinants of insomnia among a group of HCWs (15). These observations highlight the substantial impact of COVID-19 pandemic on sleep quality.

As the COVID-19 pandemic continues, providing proper strategies is of great importance to support HCWs, especially those who are involved with treating COVID-19 patients (19). Our study identified that the unavailability of PPE is a significant risk factor of both anxiety and insomnia. Therefore, the proper control of risk factors together with appropriate psychological support, in the form of counseling services, may help minimize the psychological impact of COVID-19 on HCWs.

We have encountered several limitations while conducting our study. First, our findings were based on the data derived from a cross-sectional design, and thus, making causal inferences was inapplicable. Second, the possibility of selection bias was non-negligible because all frontline workers at Jilin hospital were invited to participate in our study, and maybe those who voluntarily participated could have been more aware of their mental health issues rather than those who did not participate. Third, the generalizability of our findings was limited due to the recruitment of participants from a single Province, and since the impact of COVID-19 was different among various settings and hospitals throughout China, conflicting observations may be seen. Fourth, it has been suggested that obesity is associated with increased risk of anxiety, and this variable, in addition to other risk factors, was not studied in our research (20). Fifth, we did not include the ‘psychiatric support services' as a variable in our analysis, and thus, could not determine if anxiety was associated with those not supported during the COVID-19 pandemic. It has been reported in a large cohort study (N=20,013) that females are significantly more likely to develop anxiety compared to males (13). In our study, we could not reach such conclusion because most of the surveyed participants were females (93.88%). Therefore, more studies with larger sample sizes are still warranted to provide more evidence in this matter.

We conclude that in this survey-based study, HCWs fighting against COVID-19 at Jilin hospitals reported high rates of anxiety and insomnia. The unavailability of PPE ‘in place’ was a significant risk factor of both anxiety and insomnia. The proper address of mental health problems among medical workers is a critical component of public health measures during the COVID-19 pandemic.

## Supplementary Material

Click here to view [pdf].

## References

[1] Wang C, Horby PW, Hayden FG, Gao GF (2020). A novel coronavirus outbreak of global health concern. Lancet.

[2] Ahn DG, Shin HJ, Kim MH, Lee S, Kim HS, Myoung J (2020). Current status of epidemiology, diagnosis, therapeutics, and vaccines for novel coronavirus disease 2019 (COVID-19). J Microbiol Biotechnol.

[3] Smith N, Fraser M (2020). Straining the system: novel coronavirus (COVID-19) and preparedness for concomitant disasters. Am J Public Health.

[4] Lai J, Ma S, Wang Y, Cai Z, Hu J, Wei N (2020). Factors associated with mental health outcomes among health care workers exposed to coronavirus disease 2019. JAMA Netw Open.

[5] Pinto-Meza A, Serrano-Blanco A, Peãarrubia MT, Blanco E, Haro JM (2005). Assessing depression in primary care with the PHQ-9: can it be carried out over the telephone?. J Gen Intern Med.

[6] Raosoft Inc (2004). Sample size calculator.

[7] Shan G (2018). Sample size calculation for agreement between two raters with binary endpoints using exact tests. Stat Methods Med Res.

[8] Zung WW (1971). A rating instrument for anxiety disorders. Psychosomatics Psychiatry.

[9] Hodiamont P (1991). How normal are anxiety and fear?. Int J Soc Psychiatry.

[10] Morin CM, Belleville G, Bélanger L, Ivers H (2011). The Insomnia Severity Index: psychometric indicators to detect insomnia cases and evaluate treatment response. Sleep.

[11] Bastien CH, Valliàres A, Morin CM (2001). Validation of the Insomnia Severity Index as an outcome measure for insomnia research. Sleep Med.

[12] Huang Y, Wang Y, Wang H, Liu Z, Yu X, Yan J (2019). Prevalence of mental disorders in China: a cross-sectional epidemiological study. Lancet Psychiatry.

[13] McLean CP, Asnaani A, Litz BT, Hofmann SG (2011). Gender differences in anxiety disorders: prevalence, course of illness, comorbidity and burden of illness. J Psychiatr Res.

[14] Huang Y, Zhao N (2021). Mental health burden for the public affected by the COVID-19 outbreak in China: Who will be the high-risk group?. Psychol Health Med.

[15] Zhang C, Yang L, Liu S, Ma S, Wang Y, Cai Z (2020). Survey of insomnia and related social psychological factors among medical staff involved in the 2019 novel coronavirus disease outbreak. Front Psychiatry.

[16] Liu CY, Yang YZ, Zhang XM, Xu X, Dou QL, Zhang WW (2020). The prevalence and influencing factors in anxiety in medical workers fighting COVID-19 in China: a cross-sectional survey. Epidemiol Infect.

[17] Tan BYQ, Chew NWS, Lee GKH, Jing M, Goh Y, Yeo LLL (2020). Psychological impact of the COVID-19 pandemic on health care workers in Singapore. Ann Intern Med.

[18] Akerstedt T (2006). Psychosocial stress and impaired sleep. Scand J Work Environ Health.

[19] Tanne JH, Hayasaki E, Zastrow M, Pulla P, Smith P, Rada AG (2020). Covid-19: how doctors and healthcare systems are tackling coronavirus worldwide. BMJ.

[20] Rajan TM, Menon V (2017). Psychiatric disorders and obesity: a review of association studies. J Postgrad Med.

